# The Application of the Corn Sweat in Disease

**Published:** 1877-09

**Authors:** Frank Allport

**Affiliations:** Sycamore, Illinois


### THE APPLICATION OF THE CORN SWEAT IN DISEASE.

By Frank Allport, M. D., of Sycamore, Illinois.

In the February number of the Journal and Examiner, for 1877, appeared a short article on the "Corn Sweat." To that article I must refer those who read this, for the method of administering the "sweat," and confine myself now to its application in disease, and to a brief resumé of a few cases in which it was the chief remedial agent used.

I would not be understood to mean that the true nature of this remedy is to be found only in the "Corn Sweat." There is no particular virtue to be attributed to the corn. It is merely a simple, efficacious and convenient method of administering the principle; viz., "*external heat.*" Perhaps the chief value of external heat is in the treatment of the "pyrexiæ," and among these, it is in scarlet fever that I am more especially prepared to urge its use.

I have treated twenty-six cases of this disease, in which my main reliance was the "Corn Sweat," and have not lost a patient. Among these, only one was left with any of the troublesome sequelæ. This was a slight otorrhœa, which was easily cured in a few days, and in which the "Sweat" was not applied as soon as it should have been. No dropsy occurred in any of them. From my twenty-six cases, I have selected a few of the most typical, of which I will give a brief synopsis.

Case I. A. J., aged 10 years, taken sick Feb. 8th. I was called Feb. 10th, and found a well marked case of scarlet fever, with the exception of the eruption not having yet made its appearance. Patient was delirious; urine suppressed, temperature in axilla 107° F., pulse 144. The severity of the case was at once apparent, and the most active treatment was resolved upon. In three-quarters of an hour, the patient was in a "Corn Sweat," and had taken fifteen drops of the sweet spirits of nitre, and twenty grains of the sulpho-carbolate of sodium. In a quarter of an hour after the "sweat" was commenced, a slight moisture was felt on the surface, which in half an hour developed into a profuse perspiration. The "sweat" lasted about an hour and a half, at which time a distinct rash could be seen all over the body. The temperature was reduced to 102°, and the pulse to 140. The delirium had ceased, although perfect consciousness was not restored, and after the administration of ten drops of sweet spirits of nitre, and ten grains of sulphocorbolate of sodium, she slept three hours and awoke perfectly conscious, although much prostrated. At this time she passed about a pint and a half of very high colored urine. The eruption was now quite distinct, temp. 103°; pulse 132. The nitre and sodium were ordered, ten grains, and ten drops respectively, every four hours. Calling again in nine hours, I found the patient a little flighty. Only about a quarter of a pint of urine had been passed. Temp. 104°; pulse 136. Another "sweat" was administered, lasting about an hour and a half. Half an hour after it was finished, the temperature stood 102°, and the pulse 120. She now slept quietly six hours, and awoke perfectly conscious. About five o'clock of this day, (Feb. 11th), about 12 hours after the administration of the second "sweat," the patient was in good condition. Temp. 102°; pulse 120. About a quart of urine had been passed since two o'clock, and several smaller discharges before that time, and a free operation from the bowels had occurred. I administered another "sweat," however, which lasted for about an hour, and which reduced the temp. to 101°. She passed a quiet night. She went on to make a good recovery, and in two weeks and one day, after the attack (Feb. 8th), the rash had disappeared, the patient was up and around the house. I gave her a short "sweat" every day, until Feb. 18th. Administered the nitre and sodium till Feb. 15th, when I substituted for them iron and quinine.

Case II. B. F., aged three years, Nov. 15th, was taken sick in the afternoon. I was immediately called and found the patient, although not very sick as yet, still with some of the symptoms of scarlet fever. At any rate, as the disease was prevalent in the neighborhood, I resolved to treat the child, as if I could diagnosticate scarlet fever without any difficulty. The temperature stood 100°, and the pulse 115.

I prescribed a diuretic, and placed the patient in a "Corn Sweat," not without some trouble, however, as she was so young. I kept her there for about three-quarters of an hour, at the end of which time a faint efflorescence had appeared. The temperature was still 100° and the pulse 110. In about fifteen hours, the temperature had reached 101°, and the pulse 120. The secretion of urine had been scanty and high-colored. I placed the patient again in a "Corn Sweat," which lasted for about an hour. A very free perspiration took place, and the rash was now very bright all over the body. Half an hour after the "sweat," the temperature was 100°; pulse 110. In about ten hours I called again, and found no change. Gave another "sweat" of about half an hour's duration, which made no difference in either temperature or pulse. Between the two last visits, she had had, twice, a plentiful discharge of urine, and an injection of castor oil had produced an evacuation from the bowels. In about twelve hours, after a night of quiet sleep, the temperature was 100°, and the pulse 106. The urine was now plentiful, and the child was doing well in every respect. This was on the third day. The temperature kept steadily at 100° until the seventh day, (with the exception of one day, when it was 101°) when it began to decline, together with the pulse; and a week and a half after she was taken sick, all symptoms of the fever had disappeared. I gave her tonic treatment at the latter end of the disease, under which she made a good recovery.

The case that I am about to describe, is the severest one that came under my direct observation, and I think illustrates most fully the peculiar efficacy of this remedy in scarlet fever.

CASE III. H. C. Aged 12 years. Was taken sick, Jan. 3, with intense headache and vomiting. In a short time delirium, followed by other symptoms of the disease, set in. After the child had been in this condition for two days, during which time no physician saw her, I found her in the state mentioned above. There was a very scanty secretion of urine, and the faintest appearance of a dirty-red efflorescence, here and there on the body. The temperature in the axilla was 109°, and the pulse 156. Here was clearly a case of malignant scarlet fever. I gave ten grains of quinine, and a half a teaspoonful of sweet spirits of nitre, in a wineglassful of brandy. Put the patient into a "corn sweat," which lasted for about two hours. The rash became a trifle more visible, and the temperature was reduced one degree. These were the only changes. In two hours I repeated the operation, the temperature, in the meantime, returning to 109°; and had the satisfaction of seeing it reduced one more degree, (107°); the pulse numbered 152, and the rash rendered still more distinct. I repeated also the doses of nitre and brandy, and gave five grains of quinine. In three hours another "sweat" was given, lasting about an hour and a half; at the end of which the rash came out all over the body, in a scarlet-red hue, and in half an hour the temperature was 103°, and the pulse 140. The patient was now much more quiet, and disposed to sleep. Urine was passed, a short time after the "sweat," quite plentifully. I ordered nitre and quinine, in doses of ten drops, and two grains, respectively. In six hours after the termination of the last "sweat," the temperature having risen to 105°, and the pulse to 148, another "sweat" was given, which lasted an hour and a half, and which reduced the first to 103°, and the latter to 140. A plentiful evacuation of urine had occurred meantime, and an injection of castor oil had produced a free discharge from the bowels. The delirium had disappeared, and the eruption was decided. Ten hours from this the temperature was 105°, and the pulse 148. A "sweat" reduced these to 102° and 136 respectively. Several discharges of urine had taken place meanwhile. She was still quite rational, and slept well that night. In twelve hours she was in the same condition. The urine was somewhat scanty, and high colored. She had had a small discharge from the bowels, the temperature was 104°, and the pulse 140. Another "sweat" reduced these to 102°, and 132. She had now been sick five days, and was in a fine condition to recover. She did recover at the end of about three weeks, with a slight otorrhœa. I could enumerate many other cases of scarlet fever, in which the efficacy of the "sweat" would, I think, be quite as strikingly shown as in the foregoing.

I have two other cases to submit, one of typhoid fever and the other of dropsy, in which the "corn sweat" was used.

Case IV. A. E., aged 11 years. She had been feeling badly for some days. Had slight headache, and an indisposition to either work or play. Nov. 19th, she took to her bed with diarrhœa, slight tenderness in right iliac region and cough. Temp. 100°; pulse 99. I ordered a mixture containing sweet spirits of nitre, and syrup of ipecac; and powders of Dovers' powder and quinine. During the day her symptoms continued much the same, except that they all, with the exception of the diarrhœa, (which was alleviated) became aggravated. In the evening her temperature was 101°, and her pulse 105. One other symptom was now added to the list, viz., epistaxis.

During the night I was called and found the patient delirious. Temperature 102°; pulse 110. At about two o'clock, had her in a "corn-sweat." She soon began to prespire freely. She was kept in the "sweat" for about two hours, and *before it was completed*, her delirium ceased, and she was laughing and playing with those around her. At about six o'clock that morning, her temperature was 99°, and her pulse 100. I did not find it necessary to give another "sweat." All her untoward symptoms disappeared, and in two or three days she was up and around the house, feeling quite well considering the circumstances.

Case V. J. C., aged 5 years Nov. 24, 1876. Patient was greatly enlarged; œdema was everywhere plainly visible, and the abdomen seemed distended to its fullest capacity. Dyspnœa was very marked and vomiting had taken place.

The child took scarcely any interest in surrounding objects, and would hardly speak, but was still perfectly rational. A subsequent examination of the urine, (of which the secretion was very small), revealed the presence of albumen, renal epithelum, and epithelial casts. I put the patient in a "corn sweat" and ordered elaterium and sweet spirits of nitre, to be administered three times a day. The patient was in the "sweat" for nearly two hours, and had profuse perspiration. I neglected to measure the abdomen, but am convinced its circumference was reduced at least an inch. Another "sweat" was given that evening. I continued them once every day for three days, at which time the patient appeared to be reduced to his normal size. From that time to the present (May, 1877), no dropsical symptoms have appeared. The child is apparently well.

In scarlet fever, it cannot be in the treatment of the *disease per se*, that this measure is valuable, but *in the removal from the body of its poisonous and effete materials* confined by the nature of the affection. By its relaxing powers it compels the blood to seek the surface, at the same time relieving viscerial congestion. *In all fevers*, the muscles and other tissues waste. With the systemic waste the production of carbonic acid, urea, and other excrementitious substances, is increased, yet their means of egress are curtailed. *To meet this emergency, some way of increasing the secretion of urine and perspiration must be found. The external application of heat seems to answer this purpose. It opens the pores of the tightly closed sweat-ducts, and allows the water of the blood, loaded with the products of disease to escape, and by so doing creates an entirely new state of affairs, under which the patient is in an infinitely better condition to recover. Some diuretics, such as sweet spirits of nitre, may also be administered, but the "sweat" will so far relieve renal congestion that a freer secretion of urine will be observed.*

